# MicroRNA-30e* Suppresses Dengue Virus Replication by Promoting NF-κB–Dependent IFN Production

**DOI:** 10.1371/journal.pntd.0003088

**Published:** 2014-08-14

**Authors:** Xun Zhu, Zhenjian He, Yiwen Hu, Weitao Wen, Cuiji Lin, Jianchen Yu, Jing Pan, Ran Li, Haijing Deng, Shaowei Liao, Jie Yuan, Jueheng Wu, Jun Li, Mengfeng Li

**Affiliations:** 1 Key Laboratory of Tropical Disease Control (Sun Yat-sen University), Ministry of Education, Guangzhou, China; 2 Guangdong Province Key Laboratory of Functional Molecules in Oceanic Microorganism (Sun Yat-sen University), Bureau of Education, Guangzhou, China; 3 Department of Microbiology, Zhongshan School of Medicine, Sun Yat-sen University, Guangzhou, China; 4 Department of Clinical Medicine, Zhongshan School of Medicine, Sun Yat-sen University, Guangzhou, China; 5 Department of Biochemistry, Zhongshan School of Medicine, Sun Yat-sen University, Guangzhou, China; Florida Gulf Coast University, United States of America

## Abstract

MicroRNAs have been shown to contribute to a repertoire of host-pathogen interactions during viral infection. Our previous study demonstrated that microRNA-30e* (miR-30e*) directly targeted the IκBα 3′-UTR and disrupted the NF-κB/IκBα negative feedback loop, leading to hyperactivation of NF-κB. This current study investigated the possible role of miR-30e* in the regulation of innate immunity associated with dengue virus (DENV) infection. We found that DENV infection could induce miR-30e* expression in DENV-permissive cells, and such an overexpression of miR-30e* upregulated IFN-β and the downstream IFN-stimulated genes (ISGs) such as *OAS1*, *MxA* and *IFITM1*, and suppressed DENV replication. Furthermore, suppression of IκBα mediates the enhancing effect of miR-30e* on IFN-β-induced antiviral response. Collectively, our findings suggest a modulatory role of miR-30e* in DENV induced IFN-β signaling via the NF-κB-dependent pathway. Further investigation is needed to evaluate whether miR-30e* has an anti-DENV effect *in vivo*.

## Author Summary

Dengue is one of the most prevalent mosquito-borne viral diseases; though it is caused by the Dengue virus (DENV) in tropical/subtropical areas, it has shown tendency toward becoming a global public health concern, with estimated annual numbers of 50–100 million dengue infection cases and 500,000 people with severe disease who require hospitalization worldwide. Thus far no licensed vaccine or specific anti-DENV treatment for dengue is clinically available. Understanding the interaction of DENV with their human hosts is key to identifying potential therapeutic targets. In this work, we found that microRNA miR-30e* significantly suppressed DENV replication by promoting NF-κB-dependent IFN-β production. Our findings identifies miR-30e* as a possible restriction host factor for DENV infection, via positively modulating the antiviral innate immune response. Thus, this work broadens the understanding of the pivotal roles of miR-30e* in the interaction between DENV and the host.

## Introduction

Dengue is an important mosquito-borne viral disease affecting humans, characterized by a spectrum of symptoms ranging from relatively mild dengue fever (DF) to more severe, and commonly lethal, dengue hemorrhagic fever (DHF) and dengue shock syndrome (DSS) [1], [2]. Dengue virus (DENV), the causative agent of dengue, is a positive-polarity, single-stranded RNA virus belonging to the *Flaviviridae* family. DENV is divided into 4 antigenically related but distinct serotypes, types 1–4 (DENV1–DENV4). An estimated more than 50 million people contract dengue virus annually, leading to approximately 500,000 hospitalizations and 25,000 deaths, particularly among children [3]. Despite an urgent need for effective counter-DENV strategies, thus far neither effective vaccine nor specific antiviral treatment exists for dengue.

The host innate immune system acts as the first line of defense against viruses, and establishment of viral infection requires the pathogen to antagonize such innate immunity [4]. Type I interferons (IFNs), which mainly include IFN-α and IFN-β, are vital components of the anti-viral innate immune system. Rapid synthesis and secretion of these cytokines is critical for host cells to establish an antiviral state. The initial induction of type I interferon is dependent on the recognition and activation of pathogens by pattern-recognition receptors, which further activates transcription factors, such as NF-κB. Under basal conditions, the NF-κB is retained in the cytoplasm by IκBα, which are subject to IκB kinase (IKK)-mediated phosphorylation under stimulation, resulting in degradation of IκBα and translocation of NF-κB into the nucleus [5], [6]. Activation of NF-κB in turn leads to the gene encoding IFN-β (*Ifnb1*) transcription and IFN-β production combined with IFN regulatory factor 3 (IRF3) [7], which ultimately rendering the cell to establishment of an antiviral state by increasing a subset of IFN-stimulated genes coding antiviral proteins or microRNAs [8]. NF-κB activation is positively regulated by various signaling molecules involved in the repression of its natural inhibitors, such as IκBα. Recent study revealed that zinc finger protein ZBTB20 promotes toll-like receptor-triggered innate immune responses by repressing IκBα gene transcription [9]. Our previous work also demonstrated that miR-30e* promoted nuclear localization and activation NF-κB, via directly interacted with IκBα 3′-UTR and suppresses IκBα expression [10].

microRNAs (miRNAs) are ∼22 nucleotide (nt) short non-coding RNAs (ncRNAs) that modulate gene expression at post-transcriptional level by targeting mRNAs for degradation or by inhibiting translation [11]. Increasing evidence indicates that miRNAs are not only involved in maintenance of normal cell functions, but also participate in host-virus interactions and play a pivotal role in the regulation of viral replication [12]. For example, miR-122, a liver-specific miRNA, facilitates the replication of the viral RNA by targeting the 5′ nontranslational region of hepatitis C virus (HCV) genomic RNA [13]. In addition, miR-323, miR-491, and miR-654 are reported to inhibit replication of the H1N1 influenza A virus through binding to the PB1 gene [14]. Previous studies also provide evidence that modulating cellular miRNAs may be one of the mechanisms that interferon system combat viral infection. Wang *et.al* demonstrated that cellular inducible miR-155 feedback positively regulates host antiviral innate immune response by promoting type I IFN signaling via targeting suppressor of cytokine signaling 1 (SOCS1) [15].

In the present study, we identified that cellular miR-30e* was up-regulated by DENV infection. Further investigation indicated that miR-30e* suppressed DENV replication by promoting IFN-β production. Additionally, we found that the antiviral effect of miR-30e* is mainly dependent on targeting IκBα in DENV-permissive cells. Therefore, our data suggest that miR-30e* might be an effective approach for improvements of nucleic acid inhibitors of DENV and implies a new therapeutic strategy for DENV infection in humans.

## Materials and Methods

### Cell culture and virus

The human monocyte cell line U937 was cultured in RPMI-1640 medium (Invitrogen, Carlsbad, CA) supplemented with 10% fetal bovine serum (FBS) (GIBCO, Carlsbad, CA). The HeLa cell line was cultured at 37°C and 5% CO_2_ in Dulbecco's modified Eagle's medium (DMEM) (Invitrogen, Carlsbad, CA) supplemented with 10% FBS, 2 mM L-glutamine, 100 µg/ml streptomycin and 100 units/ml penicillin (Invitrogen, Carlsbad, CA). C6/36 *Aedes albopictus* cells (ATCC, CRL-1660) were maintained at 28°C and 5% CO_2_ in DMEM supplemented with 10% FBS. The Dengue 1 virus Hawaii strain, Dengue 2 virus New Guinea C strain and Dengue 3 virus H241 strain were kindly provided by the Guangzhou Center for Disease Control [16], [17] and propagated in the mosquito cell line C6/36. Viral stocks were stored at −80°C and titrated on C6/36 cells. For isolation of peripheral blood mononuclear cells (PBMC), whole blood was collected and subjected to Ficoll–Hypaque density gradient centrifugation according to the manufacturer's instruction (Lymphoprep kit, Nycomed, Oslo, Norway) to obtain purified PBMC [18], which were then resuspended and cultured in RPMI-1640 medium (Invitrogen, Carlsbad, CA) supplemented with 10% fetal bovine serum (FBS) (Hyclone, Logan, UT), 15 mM HEPES, 2 mM L-glutamine, 100 µg/ml streptomycin and 100 units/ml penicillin (Invitrogen, Carlsbad, CA).

### Quantitative real-time PCR (qRT-PCR)

Total RNA was extracted with Trizol reagent (Invitrogen, Carlsbad, CA) according to the manufacturer's instructions [19], [20]. For the first-strand cDNA synthesis, 500 ng of total RNA was reverse transcribed using random hexamer primer. qPCR reactions were carried out using Fast Start Universal SYBR Green Master Mix (Roche, Basel, Switzerland) and performed on Bio-Rad CFX96 real-time Detection System (Bio-Rad, Hercules, CA). All readings were normalized to the level of GAPDH mRNA. miRNA qRT-PCR was performed using the miRNA-specific TaqMan MicroRNA Assay kit (Applied Biosystems, Grand Island, NY) according to the manufacturer's instructions. miRNA expression was normalized to internal control U6 RNA. The primers sequences are shown in Supplemental Table S1.

### Cell viability analysis

Cells (1×10^4^ cells/well) in growth medium were seeded in 96-well flat-bottom plates (in triplicates), and transfected with synthetic miR-30e* mimics or negative control (NC) mimics at a final concentration of 20 nM, or a synthetic specific miR-30e* inhibitor or inhibitor negative control (inhibitor NC) at a final concentration of 50 nM, for additional 48 h. Cell viability was measured by using the MTS (3-(4,5-dimethylthiazol-2-yl)-5-(3- carboxymethoxyphenyl)-2-(4- sulfophenyl)-2*H*-tetrazolium) assay to monitor cell viability, according to the manufacturer's recommendations. Briefly, 20 µl MTS solution (CellTiter 96Aqueous One Solution reagent, Promega, Madison, WI, USA) was added to each well and incubated for an additional 4 h at 37°C. The absorbance was measured at 490 nm using a microplate reader (Bio-Tek Synergy 2, Winooski, VT, USA).

### Transient transfection and luciferase assay

The pGL3-IκBα-3′-UTR reporter plasmid was based on the pGL3 vector and described previously [10]. Cells were seeded in a 24-well plate 24 h prior to transfection, and 100 ng of pGL3-IκBα-3′-UTR reporter construct along with 10 ng of the control plasmid (pRL-TK Vector; Promega) and miRNA at indicated concentrations were cotransfected into the cells using the Lipofectamine 2000 reagent (Invitrogen, Carlsbad, CA). Twenty-four hours after transfection, the whole cell lysates were harvested and assayed with a Dual-Luciferase Reporter Assay System kit (Promega, San Luis Obispo, CA) to measure the luciferase activity according to the manufacturer's instruction. The IκBα-ORF was generated by subcloning PCR-amplified full-length human IκBα open reading frame (without 3′-UTR) into the pcDNA3.1 vector as previously described [10]. The miR-30e* mimics, negative control (NC) mimics, miR-30e* inhibitor and inhibitor negative control (Inhibitor NC) were purchased from RiBoBio (RiBoBio Inc., Guangzhou, China).

### Western blot analysis and immunofluorescence assays

Western blot analysis was performed as described previously [10], [21], using the following primary antibodies: anti-DENV antibody D1-11 (anti-DENV2 E, monoclonal) (Santa Cruz Biotechnology, Santa Cruz, CA), anti-DENV prM antibody (GeneTex, Alton Pkwy Irvine, CA), anti-actin antibody (Sigma-Aldrich, St. Louis, MO) and anti-IκBα antibody (Cell Signaling Technology, Danvers, MA). Protein bands were revealed by horseradish peroxidase-conjugated antibody and enhanced chemiluminescence using a commercial kit (Thermo Fisher Scientific, Rockford, IL) by following the manufacturer's suggested protocols. Immunofluorescence staining was carried out using anti-DENV antibody D1-11 (anti-DENV2 E, monoclonal) (Santa Cruz Biotechnology, Santa Cruz, CA) and Rhodamine-conjugated secondary antibody (Jackson ImmunoResearch Laboratories Inc, West Grove, PA), and the images were captured using the AxioVision Rel.4.6 computerized image analysis system.

### Quantification of IFN-β by ELISA

U937 and HeLa cells were seeded in a 6-well plate and transfected with the indicated miRNA (20 nM) for 24 h. The supernatants of treated cells were assayed for IFN-β protein release using the Human Interferon-β ELISA Kit (USCN Life Science, Wuhan, China) according to the manufacturer's instruction [22], [23]. Absorbance at 450 nm was read on microplate reader by using a Bio-Tek Synergy 2 microplate reader (Winooski, VT, USA).

### Statistical analysis

Results are expressed as mean ± standard deviations (SD). Statistical analyses were performed on triplicate experiments using two-tailed Student's *t* test.

## Results

### miR-30e* expression is upregulated by DENV infection

**Figure 1:**
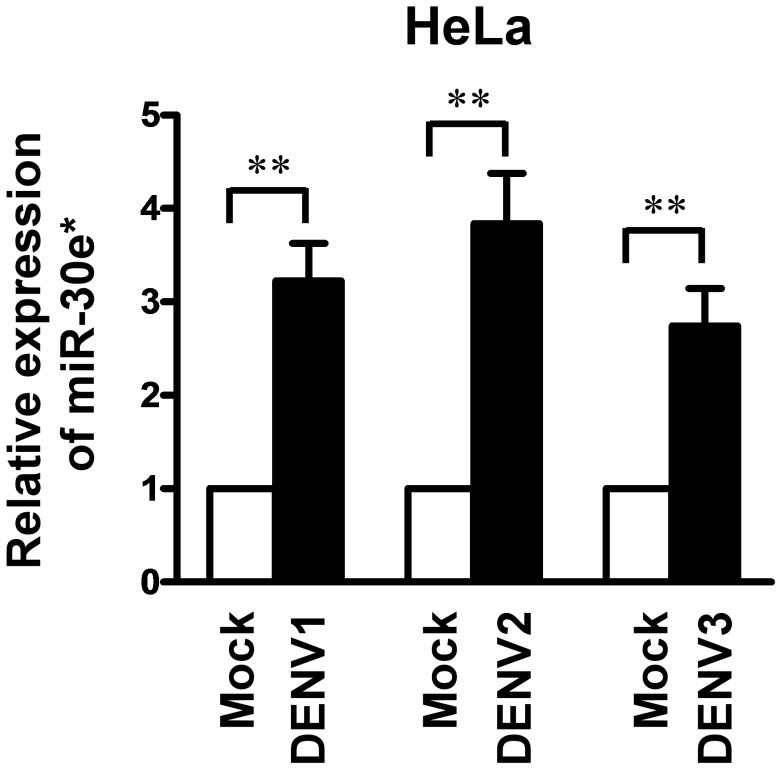
DENV infection induces miR-30e* expression. HeLa cells were infected with or without DENV1, 2 and 3 at MOI of 1 for 6 h. Expressions of miR-30e* was measured by qRT-PCR and normalized to the expression of U6 in each sample. Data show mean ± SD from three repeat experiments. **p<0.01 (Student's t-test).

To investigate the role of miR30e* in DENV infection, HeLa cells were infected with DENV1, DENV2 and DENV3, respectively, and analyzed for miR-30e* expression by real-time RT-PCR. The results showed that DENV1 infection of HeLa at MOI of 1 for 6 h led to a transcriptional induction of miR-30e* (Figure 1). Similar results were obtained when cells were infected with DENV2 and DENV3 (Figure 1). Taken together, these results suggest that expression of miR-30e* could be induced by DENV infection.

### miR-30e* suppresses the replication of DENV2

**Figure 2:**
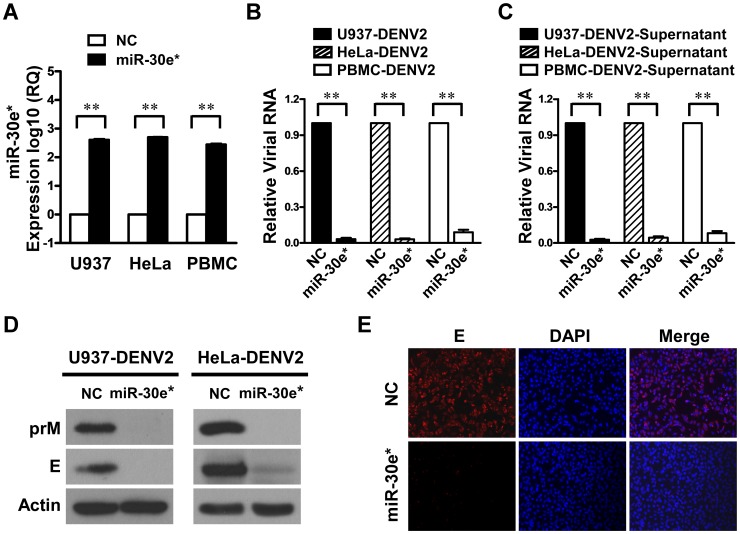
miR-30e* suppresses the replication of DENV2 in U937 and HeLa cells. U937, HeLa and PBMC cells were transfected with 20 nM of miR-30e* mimics or negative control mimics (NC) and then infected with DENV2 at MOI of 1. (**A**) The expression levels of miR-30e* were analyzed by real-time RT-PCR. The cellular viral RNA (**B**) and supernatant viral RNA (**C**) were assessed using real-time RT-PCR. Expression levels were normalized to *GAPDH*. Data show mean ± SD from three repeat experiments. **p<0.01 (Student's t-test). (**D**) miR-30e*-transfected U937 and HeLa cells were infected with DENV2, and then the viral E, prM and Actin proteins were detected by immunoblotting analysis. (**E**) miR-30e*-transfected HeLa cells were infected with DENV2, and then stained with anti-E antibody as well as DAPI, and subsequently a secondary antibody conjugated to Rhodamine was used to visualize stained E proteins. Samples were inspected by fluorescence microscope at a magnification of 200×.

We next examined whether miR-30e* has any effect on DENV replication. U937, HeLa or PBMC cells were transfected with synthetic miR-30e* mimics or negative control (NC) mimics. As shown in supplemental Figure S1A, our results revealed no or little inhibitory effects of miR-30e* or NC mimics on either tested cell lines or primary cells at dose of 20 nM. At 24 h after transfection, cells were challenged with DENV2 at MOI of 1, and cellular and supernatant viral RNA was collected quantified by real-time PCR. miR-30e* overexpression in both cell lines was verified by real-time RT-PCR (Figure 2A). Our results showed that miR-30e* caused a significant reduction of DENV2 RNA in U937, HeLa, and PBMC cells (Figure 2, B and C). Moreover, at the protein level, immunoblotting analysis showed that the expressions of DENV2 prM and envelop protein (E) were markedly suppressed by miR-30e* (Figure 2D), and staining experiments revealed results in accordance with those of the immunoblotting analysis (Figure 2E), suggesting a potent inhibitory effect of host miR-30e* on DENV2 RNA and protein synthesis.

### Silencing miR-30e* enhances virus replication

**Figure 3:**
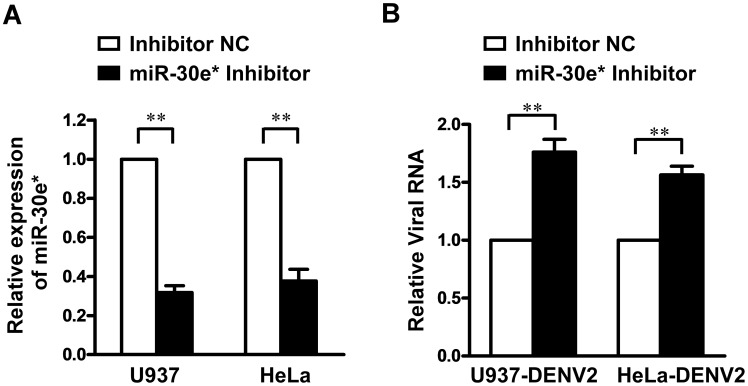
miR-30e* inhibition increases DENV2 replication. (**A**) U937 and HeLa cells were transfected with a specific miR-30e* inhibitor or negative control (Inhibitor NC) at a final concentration of 50 nM. The expression levels of miR-30e* were analyzed by real-time RT-PCR. (**B**) Prior transfection of U937 and HeLa cells with miR-30e* inhibitor accelerated DENV2 replication. DENV2 RNA levels were measured by real-time RT-PCR. Data show mean ± SD derived from three repeat experiments. **p<0.01 (Student's t-test).

To further investigate whether endogenous miR-30e* was involved in modulating virus replication, U937 and HeLa cells were transfected with a synthetic specific miR-30e* inhibitor or inhibitor negative control (Inhibitor NC). As shown in supplemental Figure S1B, our results also revealed no or little inhibitory effects of miR-30e* inhibitor or inhibitor NC on either U937 or HeLa cell line at dose of 50 nM. At 24 h after transfection, cells were challenged with DENV2 at MOI of 1, and the cellular viral RNA was quantified by real-time RT-PCR. miR-30e* repression in both cell lines was verified by real-time RT-PCR (Figure 3A). As shown in Figure 3B, miR-30e*-inhibited cells exhibited increased replication of DENV2. These results indicated that endogenous miR-30e* functions to suppress DENV2 propagation.

### miR-30e* represses DENV2 replication by promoting IFN-β production

**Figure 4:**
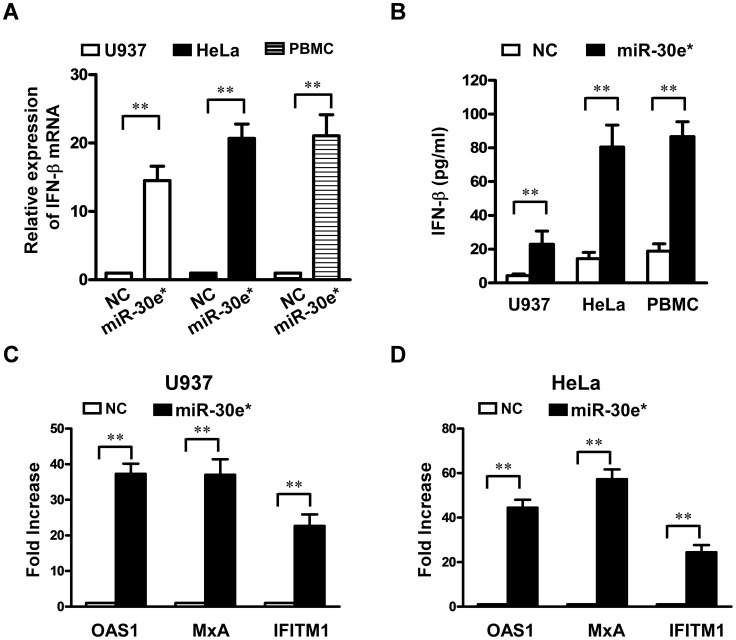
miR-30e* enhances expression of IFN-β. U937, HeLa and PBMC cells were transfected with 20 nM of miR-30e* mimics or negative control mimics (NC) and then infected with DENV2 at MOI of 1. (**A**) mRNA levels of IFN-β were measured by real-time RT-PCR. (**B**) Protein levels of IFN-β were determined by ELISA. Expression of IFN-induced genes *OAS1*, *MxA* and *IFITM1* in U937 cells (**C**) and HeLa cells (**D**) were determined by real-time RT-PCR at 24 h after transfection. Data show mean ± SD derived from three repeat experiments. **p<0.01 (Student's t-test).

As type I IFN plays a pivotal role in the host antiviral innate immune response, we wondered whether elevated IFN production was responsible for the inhibition of virus replication in miR-30e*-overexpressing cells. Our results showed that miR-30e* significantly induced mRNA and protein expression of IFN-β in U937, HeLa and PBMC cells (Figure 4, A and B). Furthermore, IFN-inducible genes, including *OAS1*, *MxA* and *IFITM1*, were induced by miR-30e* transfection in both U937 and HeLa cells (Figure 4, C and D). These data suggested that miR-30e* suppression of DENV2 infection was closely associated with IFN-β production.

### The antiviral role of miR-30e* is mediated by targeting IκBα

**Figure 5:**
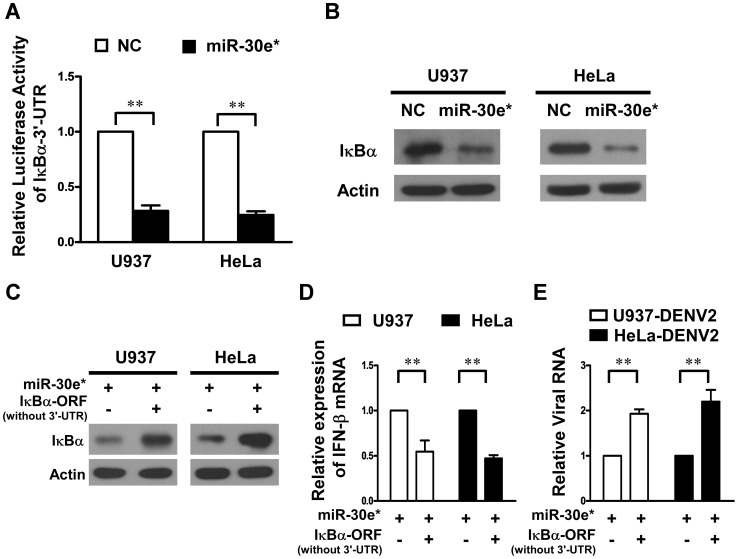
IκBα is a direct target gene of miR-30e*. (**A**) U937 and HeLa were cotransfected with 100 ng of the indicated pGL3-IκBα-3′-UTR reporter plasmid and 10 ng of pRL-TK plasmid (internal control), together with the negative control or miR-30e* mimics at a final concentration of 20 nM. After 24 h, firefly luciferase activity was measured and normalized to Renilla luciferase activity. (**B**) U937 and HeLa cells were transfected with 20 nM of miR-30e* mimics or negative control mimics (NC), and then the expression levels of IκBα was measured by Western blotting. (**C**) U937 and HeLa cells were cotransfected with miR-30e* mimics and pcDNA3 vector or pcDNA3- IκBα ORF (without 3′-UTR). After 24 h, IκBα and Actin were detected by Western blotting. Cells cotransfected with miR-30e* mimics along with pcDNA3 vector or pcDNA3- IκBα ORF (without 3′-UTR) as indicated for 24 h were infected with DENV2 at MOI of 1. Followed by an additional infection for 24 h, intracellular mRNA levels of IFN-β (**D**) and viral RNA (**E**) in U937 or HeLa cells were measured by real-time RT-PCR. Data show mean ± SD derived from three repeat experiments. **p<0.01 (Student's t-test).

As NF-κB pathway plays an important role in regulating IFN-β production, and our previous studies showed that miR-30e* could activate NF-κB by directly targeting the IκBα 3′-UTR [10], to further investigate the underlying mechanisms responsible for the elevated IFN-β production induced by miR-30e*, we then investigated whether miR-30e* could promote IFN-β production by activating NF-κB. We used the luciferase reporter plasmid containing the 3′-UTR sequences of IκBα mRNA and determined whether miR-30e* could directly target the 3′-UTR sequences in U937 and HeLa cells. In consistence with our previous finding with glioma cells, the results showed that cotransfection of miR-30e* was able to inhibit the luciferase reporter activity (Figure 5A). Furthermore, the protein expression of endogenous IκBα was significantly repressed by miR-30e* in both U937 and HeLa cells (Figure 5B).

In order to understand the role of IκBα in miR-30e*-induced antiviral effect, we studied the effect of ectopically overexpression of IκBα ORF (without 3′-UTR) in miR-30e*-overexpressed cells, and found that it could significantly restore the cellular IκBα protein level (Figure 5C). Our data also indicated that concomitant overexpression of the IκBα ORF (without 3′-UTR) and miR-30e* in U937 and HeLa cells resulted in suppressed IFN-β production (Figure 5D) and robustly abrogated enhanced antiviral effect of miR-30e* (Figure 5E), suggesting that miR-30e* directly targets the 3′-UTR sequences of IκBα, thus enhancing IFN-β production and suppressing DENV replication.

## Discussion

Effective activation of antiviral innate immune responses is essential for the host antiviral defense, which is tightly regulated by a variety of molecular regulators, including miRNAs. Recent evidence indicates that some viruses encode miRNAs that dampen host antiviral immunity, and on the other hand, cellular miRNAs coded by the host can be antiviral via targeting host genes or viral coding sequences [24]. In this work, we report that in DENV-infected cells, inducible miR-30e* restores IFN-β production and inhibits DENV replication, presumably through targeting IκBα and subsequent activation of NF-κB. Our study identifies miR-30e* as a possible restriction host factor for DENV infection via positively modulating the antiviral innate immune response. Further *in vivo* studies will be required to determine the potential clinical significance of the proposed role of miR-30e* in modulating host cell response to DENV infection, although it remains highly challenging to establish an applicable animal model mimicking DENV pathogenesis, especially immunopathogenesis [25], [26].

Previous studies have indicated that miR-30e* might be a multifunctional microRNA. It is likely miR-30e* might be involved in maintenance of physiological conditions such as heart development [27] and adipogenesis [28], as well as in pathogenesis of diseases such as neural tube defects [29], cancer [10], [30]–[33] and trauma [34]). It was previously shown that the expression levels of miR-30 family were higher in PBMCs collected from patients with chronic hepatitis C compared with those from healthy individuals [35]. Additionally, Pedersen *et.al* reported that the miR-30 family could be induced by type I IFN in Huh7 cells and primary hepatocytes [36]. However, the functions of miR-30 and the underlying molecular mechanism of this process remain unclear. The key finding of our present study is the identification of inducible miR-30e* in the modulation of DENV multiplication by restoring the innate immune response via activating NF-κB signaling. Since it is not yet totally clear how miRNA is involved in the intrinsic immune system's neutralization of virus threat, this work provides an example of possible mechanisms via which a host cell miRNAs participate in modulating DENV-triggered innate immunity. As IFNs are main mediators of the host antiviral defense system, it would be interesting and important to illustrate the significance of miR-30e* for other viruses, which is under active investigation in the laboratory.

Host innate immunity is the first line of antiviral defense, functional to recognize viral components and produce type I IFN and other proinflammatory cytokines. Type I IFN is extensively employed in clinical therapy of viral infection. However, the efficacies of IFN therapy vary with different viruses, disease stages and the other host factors that influence host responses to IFN [37]. The long co-evolutionary history of viruses and their hosts leads to co-development of the antiviral capability of hosts and counter-antiviral strategies of viruses. Previous reports have demonstrated that DENV is usually a weak inducer of type I IFN responses [16]. It has been recognized that DENV-encoded nonstructural protein NS2B3 physically targets human mediator of IRF3 activation (MITA), and the interaction and cleavage of MITA could block IFN production and subverts the host innate immunity [38]. Our current results extend previous investigations into the modulation of the IFN system and specifically, the ability of a host cellular miRNA, namely, miR-30e*, to upregulate and restore type I IFN production. While our current study has shown that miR-30e* might be a physiologically relevant regulator of IFN function in response to DENV infection, further investigation is needed in the future to evaluate whether it can be of therapeutic significance in the context of *in vivo* DENV infection. And it requires to be clarified whether promotion of NF-κB dependent innate immunity against DENV infection could simultaneously cause immunopathologic events associated with cytokine overproduction. Furthermore, it is also of note that by showing that viral replication remained relatively unchanged after miRNA and siRNA production was globally abrogated through knocking down Dicer, Bogerd *et al* recently reported that many viruses are refractory to miRNA or small interfering (siRNA) modulation in the host cells [39], [40]. This study is important because it raises the question whether application of miRNA- or siRNA-based strategies could be therapeutically effective in suppressing viral infection in the clinic. On the other hand, however, as the above study was performed via knocking out Dicer and global endogenous miRNA/siRNA production, it remains to be clarified whether using a high dose, exogenous miRNA could be effective to suppress DENV infection *in vivo*.

Taken together, our study shows that miR-30e*, as a positive regulator, participates in antiviral innate immune responses once induced in cells upon DENV challenge. This work broadens the understanding of the roles of miR-30e* in the interaction between host and DENV, and further studies to decipher the biological basis for the antiviral activities of miR-30e* will be of theoretical as well as practical importance in developing useful antiviral strategies.

## Supporting Information

Figure S1 Effect of miR-30e* expression and inhibition on the viability of U937, HeLa and PBMC cells. (**A**) Effects of miR-30e* or NC mimics on the viability of U937, HeLa and PBMC cells at dose of 20 nM. (**B**) Effects of miR-30e* inhibitor or inhibitor NC on the viability of U937, HeLa and PBMC cells at dose of 50 nM. Numbers of viable cells were determined by MTS assay. Data points are presented as means ± SD of triplicated experiments.
(TIF)

Table S1 **Sequences of real-time RT-PCR primers used.**
(DOC)
