# Supplementary figures and images for: MicroRNA-30e* Suppresses Dengue Virus Replication by Promoting NF-κB–Dependent IFN Production
Source: PLoS Negl Trop Dis. 2014 Aug 14;8(8):e3088. doi: 10.1371/journal.pntd.0003088 (PMC4133224; doi:10.1371/journal.pntd.0003088)

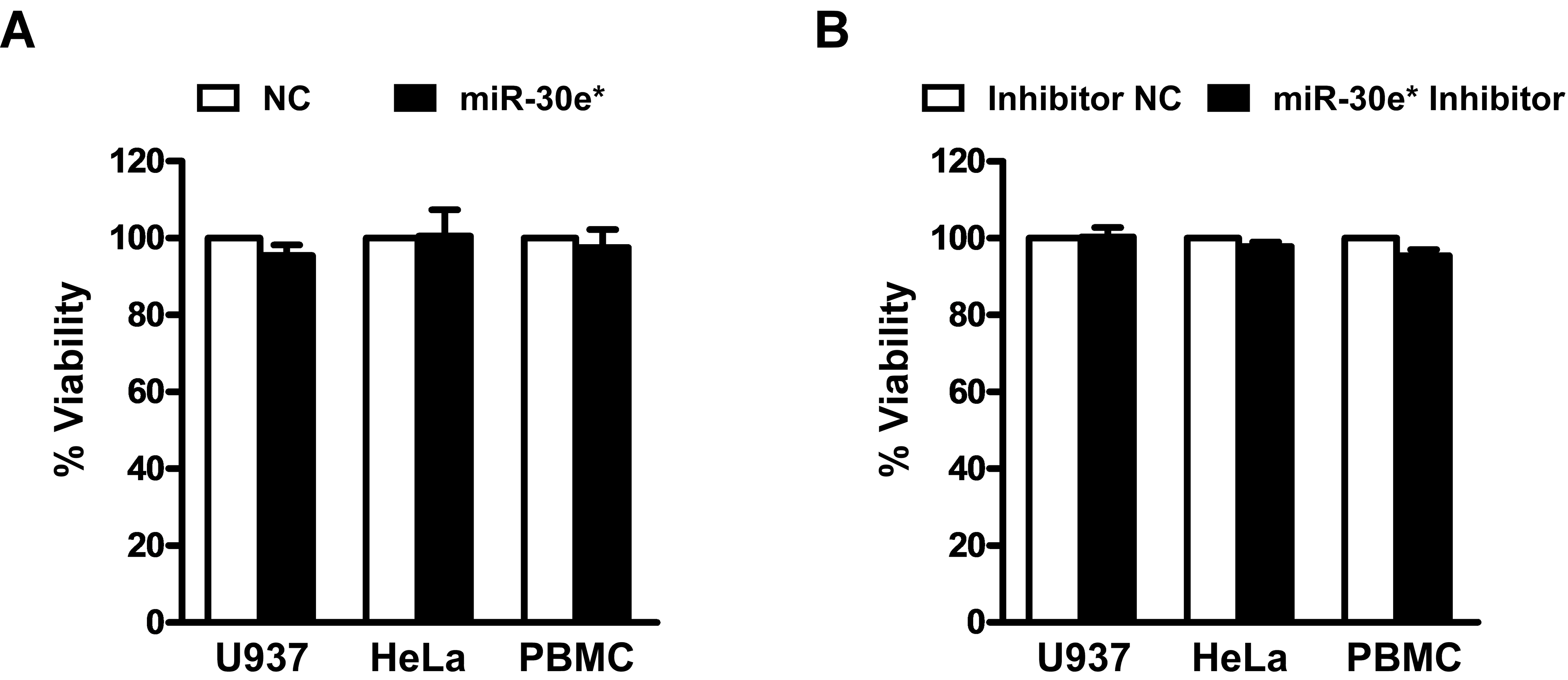

Supplement: Figure S1 — Effect of miR-30e* expression and inhibition on the viability of U937, HeLa and PBMC cells. (A) Effects of miR-30e* or NC mimics on the viability of U937, HeLa and PBMC cells at dose of 20 nM. (B) Effects of miR-30e* inhibitor or inhibitor NC on the viability of U937, HeLa and PBMC cells at dose of 50 nM. Numbers of viable cells were determined by MTS assay. Data points are presented as means ± SD of triplicated experiments. (TIF) [file pntd.0003088.s001.tif]
