# Supplementary material for: MicroRNA-30e* Suppresses Dengue Virus Replication by Promoting NF-κB–Dependent IFN Production
Source: PLoS Negl Trop Dis. 2014 Aug 14;8(8):e3088. doi: 10.1371/journal.pntd.0003088 (PMC4133224; doi:10.1371/journal.pntd.0003088)
Supplement: Table S1 — Sequences of real-time RT-PCR primers used. (DOC) [file pntd.0003088.s002.doc]

**Table S1. Sequences of real-time RT-PCR primers used**

| Target | Primers | Sequence (5’-3’) |
| --- | --- | --- |
| *DENV2* | Forward | TTATCAGTTCAAAATCCAATGTTGGT |
|  | Reverse | AGGAGGAAGCTGGGTTGACA |
|  | Probe | FAM-CATCGTCGTCACACAGCTTCCATGTTC-BHQ1 |
| *GAPDH* | Forward | GACTCATGACCACAGTCCATGC |
|  | Reverse | AGAGGCAGGGATGATGTTCTG |
|  | Probe | FAM-CATCACTGCCACCCAGAAGACTGTG-BHQ1 |
| *IFNB* | Reverse | AGTTGTTTAAACTCAGACATCAGGAGGTGCTGGG |
|  | Forward | CATTACCTGAAGGCCAAGGA |
| *MxA* | Forward | AACAACCTGTGCAGCCAGTA |
|  | Reverse | AAGGGCAACTCCTGAGAGTG |
| *OAS1* | Forward | CCAAGCTCAAGAGCCTCATC |
|  | Reverse | GAGCTCCAGGGCATACTGAG |
| *IFITM1* | Forward | TCATCCTGTCACTGGTATTCGGCTC |
|  | Reverse | GTGGGTATAAACTGCTGTATCTAGGG |
| *GAPDH* | Forward | CCACTCCTCCACCTTTGAC |
|  | Reverse | ACCCTGTTGCTGTAGCCA |
